# Diurnal rhythm in chimeric antigen receptor T cell effectiveness in an observational study of 715 patients

**DOI:** 10.1172/jci.insight.201159

**Published:** 2025-12-18

**Authors:** Patrick G Lyons, Emily Gill, Prisha Kumar, Melissa Beasley, Brenna Park-Egan, Zulfiqar A. Lokhandwala, Katie M. Lebold, Brandon Hayes-Lattin, Catherine L. Hough, Nathan Singh, Guy Hazan, Huram Mok, Janice M. Huss, Colleen A. McEvoy, Jeffrey A. Haspel

**Affiliations:** 1 Division of Pulmonary, Allergy, and Critical Care Medicine, and; 2 Knight Cancer Institute, Oregon Health & Science University, Portland, Oregon, USA.; 3 Division of Pulmonary and Critical Care Medicine,; 4 Division of Oncology, Section of Cellular Therapy, and; 5 Center for Gene and Cellular Immunotherapy, Washington University School of Medicine, St. Louis, Missouri, USA.; 6 Faculty of Health Sciences, Ben Gurion University, Beer Sheva, Israel.; 7 Pediatric Pulmonary Unit, Saban Children Hospital, Soroka University Medical Center, Beer Sheva, Israel.

**Keywords:** Clinical Research, Immunology, Oncology, Cancer immunotherapy

## Abstract

**BACKGROUND:**

Chimeric antigen receptor (CAR) T cells are a leading immunotherapy for refractory B cell malignancies; however, their effect is limited by toxicity and incomplete efficacy. Daily (circadian) rhythms in immune function may offer a lever to boost therapeutic success; however, their clinical relevance to CAR T cell therapy remains unknown.

**METHODS:**

We retrospectively analyzed CAR T cell survival and complications based on infusion time at 2 geographically distinct hospitals: Washington University School of Medicine in St. Louis, Missouri, USA (n = 384) and Oregon Health & Science University in Portland, Oregon, USA (n = 331) between January 2018 and March 2025. The primary outcome was 90-day overall survival (OS). Secondary outcomes included event-free survival (EFS), cytokine release syndrome (CRS), immune cell–associated neurotoxicity syndrome (ICANS), ICU admission, shock, respiratory failure, and infection. We quantified the independent relationship between infusion time and outcomes using multivariable mixed-effects logistic regression and time-to-event models, adjusting for patient, oncologic, and treatment characteristics.

**RESULTS:**

The therapeutic index of CAR-T cells inversely correlated with the timing of administration, with later infusions associated with lower effectiveness and more adverse outcomes. For each hour that CAR T cell treatment was delayed, the adjusted odds ratio (aOR) of 90-day mortality increased by 24% (aOR 0.76; 95% CI 0.64–0.88, *P* = 0.001), severe neurotoxicity by 17% (*P* = 0.023), and mechanical ventilation by 27% (*P* = 0.026). These temporal patterns were most pronounced in patients receiving CD19-targeting CAR T cell products. In contrast, we did not find an association between infusion time and severe CRS (aOR 0.99; 95% CI, 0.75–1.27; *P* = 0.92).

**CONCLUSION:**

Time of day is a potent and easily modifiable factor that could optimize CAR T cell clinical performance.

## Abstract 2

We report a connection between the time of day when a cancer immunotherapy called CAR T-cells is administered to patients and its effectiveness.

## Introduction

Chimeric antigen receptor (CAR) T cell therapies are a mainstay treatment for refractory B cell malignancies and an emerging option in solid tumors and autoimmune diseases (1). CAR T cells can deliver high response rates and durable remission; however, unmodifiable patient characteristics like age, performance status, and disease burden can mitigate CAR T cell clinical effectiveness (2). These same factors affect the risk of life-threatening complications of CAR T cell therapy, including cytokine release syndrome (CRS) and immune effector cell–associated neurotoxicity syndrome (ICANS) (3). To improve the clinical utility of CAR T cells, we must identify easily modifiable factors that can optimize their therapeutic index.

One candidate factor is the time of day, which reflects circadian variation in the patient’s immune system (4). Circadian rhythms are 24-hour biological cycles generated by a conserved transcription factor network called the molecular circadian “clock.” Clinical evidence and preclinical animal models indicate that the clock imposes daily rhythms on fundamental immune processes, leading immune-based therapies like vaccines, checkpoint inhibitors for cancer, and hematopoietic stem cell transplants (HSCT) to perform differently based on when they are administered (5–8). Recently, a preclinical study found that CAR T cells administered to mice at the end of their activity period (corresponding to evening in humans) fail to inhibit tumor growth (9). However, the translatability of these observations to real-world clinical practice remains unknown, both in terms of CAR T cell efficacy and toxicity. We therefore studied the relationship between the time of CAR T cell infusion and patient outcomes.

## Results

**Figure 1:**
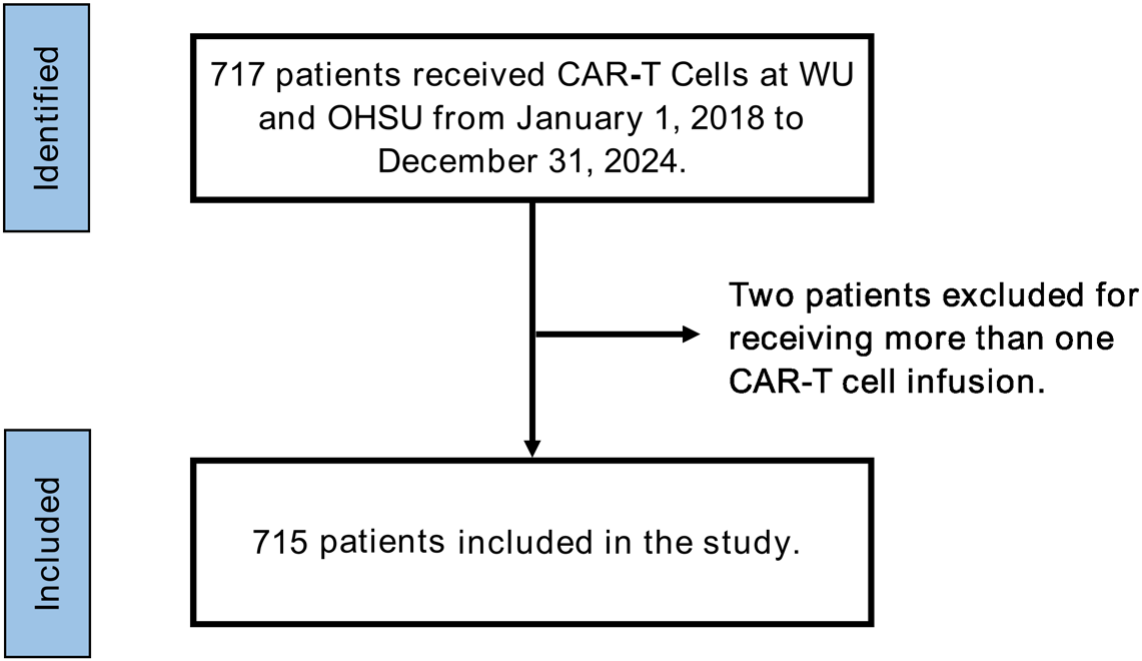
Study inclusion CONSORT Diagram.

**Figure 2:**
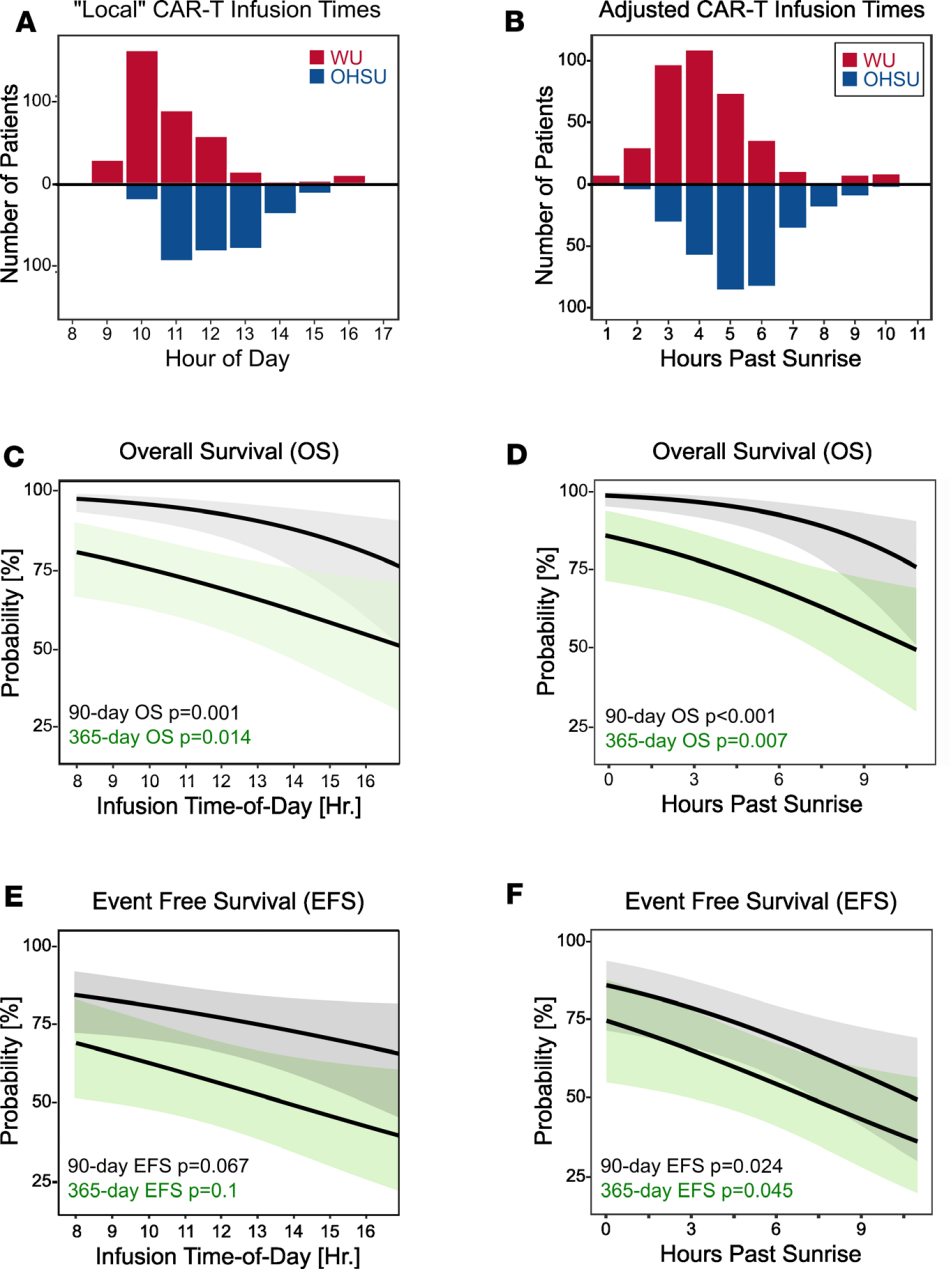
CAR-T cell treatment times vary and are associated with survival. (**A** and **B**) The distribution of CAR-T cell infusion times, expressed in terms of local clock time (**A**), or relative to local sunrise (**B**). Red bars, WU cohort (*n* = 384); blue bars, OHSU (*n* = 331). (**C** and **D**) A multivariable mixed-effects logistic regression analysis relating CAR-T infusion time, in terms of clock time (**C**) or sunrise adjusted time (**D**) to overall survival (OS) ± 95% CIs. Black line, 90-day OS; green shaded line; 365-day OS. (**E** and **F**) A multivariable mixed-effects logistic regression analysis relating CAR-T infusion time, in terms of clock time (**E**) or sunrise adjusted time (**F**) to event-free survival (EFS) ± 95% CIs. Black line, 90-day EFS; green shaded line; 365-day EFS. Wald test *P* values for trend are depicted in **E** and **F**. See Supporting Data Values file for a tabular presentation of these data.

The study included all patients receiving a single dose of CAR T cells as standard-of-care treatment between January 1, 2018, and March 31, 2025 (*n* = 715), at 2 geographically distinct quaternary-care centers: Washington University in St. Louis, Missouri, USA (WU, *n* = 384), and Oregon Health & Science University in Portland, Oregon, USA (OHSU, *n* = 331) (Figure 1 and Supplemental Table 1; supplemental material available online with this article; https://doi.org/10.1172/jci.insight.201159DS1). The cohorts had modest differences in patient characteristics, case-mix, and product selection, but both sites experienced similar rates of survival and toxicities (i.e., grade 3/4 CRS or ICANS). Infusions at WU occurred slightly earlier and spanned a broader time frame than at OHSU, whether expressed as local “clock” time (Figure 2A) or relative to the time of sunrise, which compensates for differences in latitude between the 2 centers, seasonal variation in photoperiod, and daylight saving time (Figure 2B and Supplemental Figure 1).

**Figure 3:**
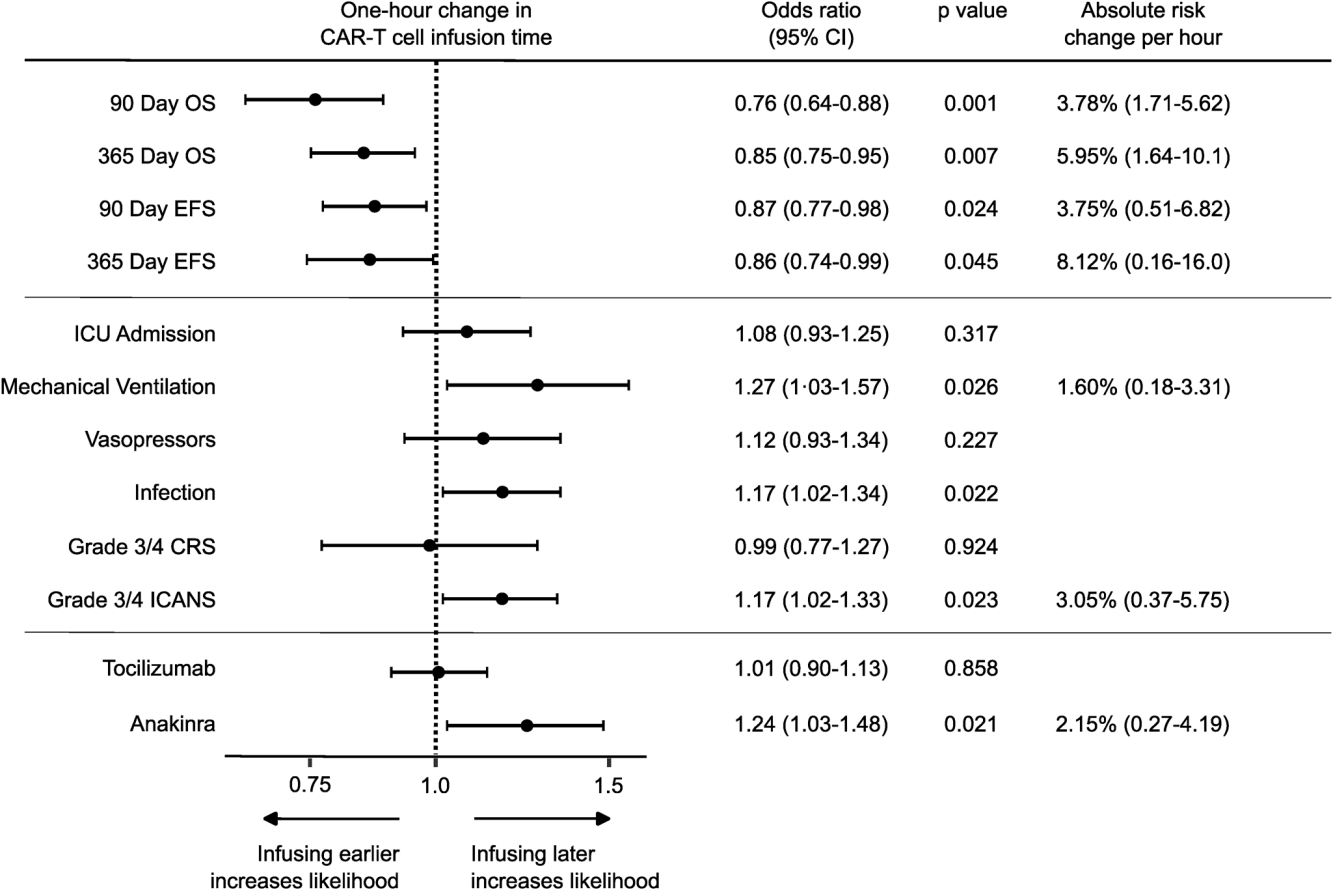
A 1-hour shift in CAR-T cell infusion time significantly correlates with effectiveness and safety. The graph depicts hourly adjusted odds ratios ± 95% CIs estimated by multivariable mixed-effects logistic regression for the indicated outcomes. The area left of the dashed line signifies that an event would be more likely to happen if CAR-T cell infusions were shifted one hour earlier; to the right of the dashed line indicates increased odds of the event happening if CAR-T cell infusions were shifted 1 hour later. See Supplemental Table 3 for a tabular presentation of these data.

To map the independent relationship between time of day and CAR T cell outcomes, we used mixed-effects multivariable logistic regression, treating infusion time as a continuous variable and adjusting for key patient, oncologic, and treatment characteristics (Figure 2, C–F, and Supplemental Table 2). These multivariable models demonstrated an inverse relationship between CAR T cell infusion time and both overall survival (OS) and event-free survival (EFS) at 90 and 365 days (Figure 2, C and E). We also observed temporal patterns for certain adverse events, including infection, acute respiratory failure requiring mechanical ventilation, and severe ICANS; interestingly, however, this was not the case for CRS (Supplemental Figure 2). As would be expected for circadian rhythms, which are strongly synchronized by light onset, the trends were statistically stronger when expressing CAR-T infusion times relative to sunrise as opposed to local clock time (Figures 2, D and F). To clarify the practical implications of these findings, we used the models to estimate the relationship between each 1-hour shift in CAR T cell infusion time and outcomes, expressed in sunrise-adjusted hours (Figure 3 and Supplemental Table 3). For each additional hour after sunrise, the adjusted odds of 90-day mortality after CAR-T cell treatment rose on average by 24% (*P* < 0.001), 365-day mortality by 15% (*P* < 0.001), grade 3–4 ICANS by 17% (*P* = 0.028), anakinra prescription (to treat ICANS) by 24% (*P* = 0.021), and mechanical ventilation by 27% (*P* = 0.026). We also used the models’ marginal effects to estimate the absolute risk reduction associated with moving CAR T cell infusions earlier in the day—analogous to established hospital quality metrics, such as time-to-antibiotics for septic shock or time-to-revascularization for myocardial infarction. Scheduling earlier CAR-T cell infusions correlated with striking benefits, on the order of 1.6%–8.1% per hour (number needed to treat, 12–65 infusions) depending on the outcome (Figure 3).

We took several steps to assess the robustness of these observations. First, we calculated E-values (evidence for causality estimates, see Methods), which quantify the minimum strength of unmeasured confounding (as an adjusted OR for a hypothetical unmeasured confounder) required to explain away the observed findings (Figure 3 and Supplemental Table 3). In multivariable models for landmarked survival, the corresponding E-values for the main effect estimates ranged from 1.35 to 1.57, indicating that moderate to strong unmeasured confounding would be required to negate the observed associations. Second, we estimated how much infusion time influences survival by comparing observed 90-day OS to the predictions of a logistic regression model that excluded CAR T cell infusion time as a covariate (Supplemental Figure 3). Throughout the day, the observed survival varied by about 10% from what would be predicted based solely on patient factors, with morning infusions exceeding the model’s predictions and evening infusions falling short. A deviation of 10% is likely clinically significant, considering the overall 90-day mortality rate for the cohort is 12.4% (Supplemental Table 1). Third, we performed a sequential “leave one out” sensitivity analysis by excluding infusions occurring in each specific hour of the day in turn (Supplemental Table 4). The inverse association between CAR T cell infusion time and 90-day OS remained consistent in this analysis, suggesting that no single time block accounts for our findings. Fourth, we manually reviewed the first consecutive 69 charts of patients who died within 90 days (*n* = 34 WU, *n* = 35 OHSU) to identify causes of death. Supplemental Table 5 shows these causes of death, which were consistently attributed to malignancy or treatment complications regardless of CAR-T cell infusion time. Fifth, we examined the CAR-T cell “post-thaw hold” duration as a possible reason for different clinical outcomes, defined as the time between when CAR-T cells were thawed and when they were infused into the patient (Supplemental Figure 4). We found no correlation between the time of day that patients received CAR-T cells and the duration of the post-thaw hold. Sixth, we manually reviewed the medical records for all CAR T cell infusions performed after 15:00 to identify potential unmeasured confounding by indication (i.e., a factor that may cause late-day infusions and also increase the risk of adverse outcomes). In these reviews (Supplemental Table 6), patients either had no clear rationale or a benign rationale for late-day infusion.

**Figure 4:**
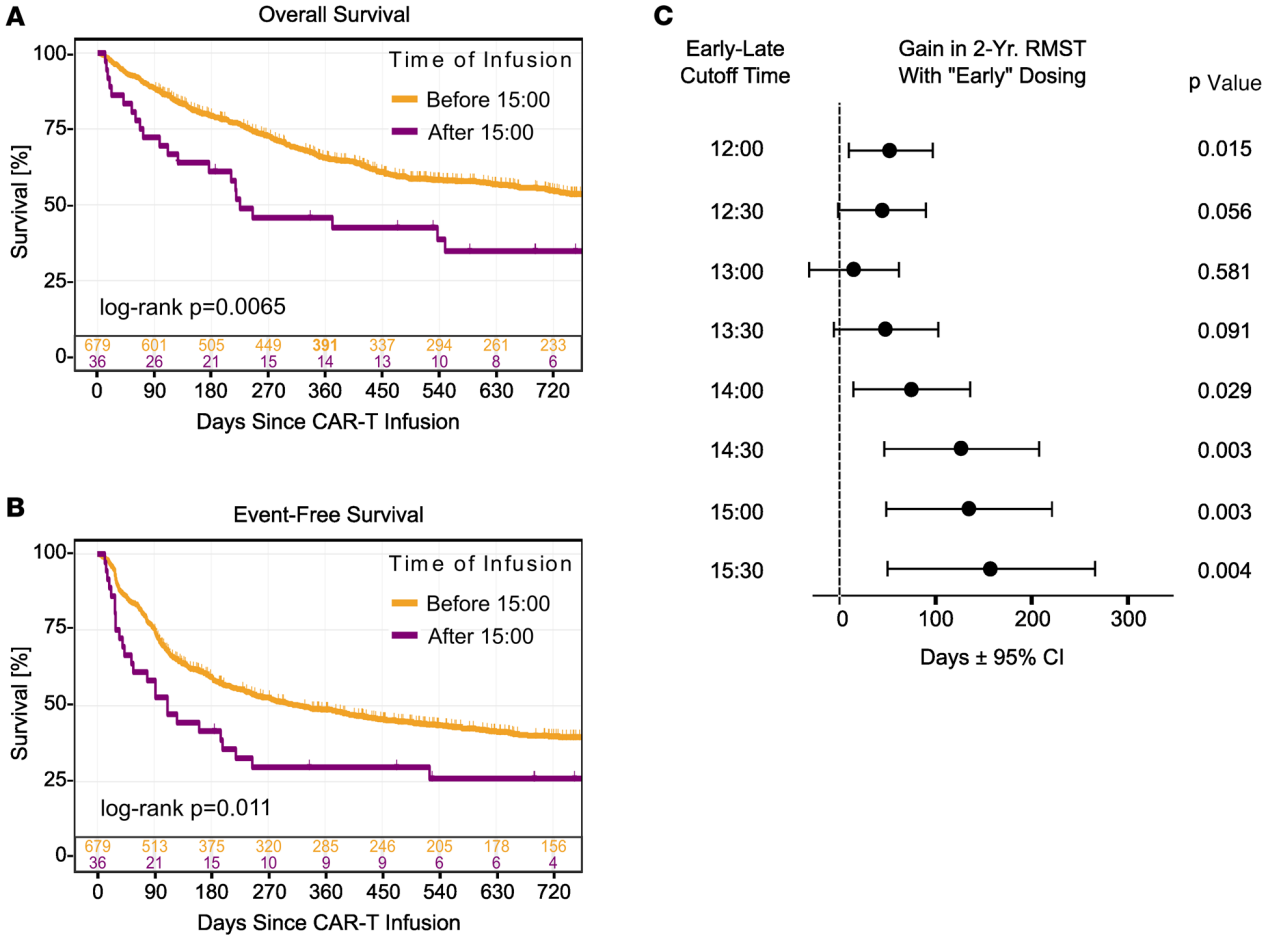
Time-to-event analyses validate time-of-day as significant variable in CAR-T cell clinical performance. (**A** and **B**) Unadjusted Kaplan-Meier analyses comparing CAR-T cell infusion times before 15:00 (orange lines, *n* = 679) to after 15:00 (purple lines, *n* = 36). (**A**) Overall survival (OS). (**B**) Event-free survival (EFS). The log-rank test *P* value is depicted. (**C**) The gain in covariate adjusted 2-year restricted mean survival time (RMST) with early day CAR-T infusions, modeling a range of cutoff times between “early” and “late.” Data are shown as ± 95% CIs. Wald’s *P* values are depicted. See Supplemental Table 7 and the Supporting Data Values file for a tabular presentation of these data.

Finally, to orthogonally validate our findings, we performed time-to-event analyses comparing survival in patients dichotomously grouped as receiving “early-day” or “late-day” infusions (Figure 4). As a starting point, we chose 15:00 as a cutoff between early and late based on practical relevance (midpoint of afternoon clinic schedule) and consistency with studies of diurnal rhythms in checkpoint inhibitors (5). Patients receiving early and late infusions had similar characteristics (Table 1). Still, those receiving later infusions exhibited worse OS and EFS, as well as more frequent complications like ICU admission and anakinra prescription for severe ICANS. (Figure 4, A and B, and Table 2). In adjusted time-to-event models (controlling for the same variables in the logistic regression approach), infusions earlier in the day increased the 2-year restricted mean survival time (RMST), particularly once the cutoff between “early” and “late” day infusions approached 14:00 (Figure 4C and Supplemental Table 7). When the time-of-day exposure was modeled as a continuous variable rather than dichotomously, each hour delay in infusion time was associated with an average reduction of 19 days in 2 year overall RMST (95% CI 6.1 to 32-day reduction) and a similar decrease in event free RMST (18-day de-crease per hour, 95% CI 3.9 to 34-day reduction). These values represent absolute differences of approximately 3% per hour, a magnitude resembling that found in logistic regression analyses (Figure 3). Altogether, CAR T cell effectiveness and toxicity varied with infusion time, with better outcomes occurring with earlier-day infusions.

Clinicians typically measure blood cell counts and serologic markers of inflammation after CAR T cell treatments. Therefore, we investigated whether standard biomarkers would be sensitive to CAR T cell infusion time. The recovery rate in absolute lymphocyte count (ALC), a predictor of CAR T efficacy (10), did not differ based on infusion time through day 14 (Supplemental Figure 5). Inflammatory serologies — CRP, LDH, and ferritin — were available for most patients (*n* = 661), and the OHSU cohort had measurements of circulating cytokines (*n* = 167). We did not detect a significant relationship between CAR-T infusion times and these laboratory test values when time was expressed as a continuous variable (Supplemental Figure 6) or when dichotomously comparing early and late infusion times (Supplemental Table 8).

**Figure 5:**
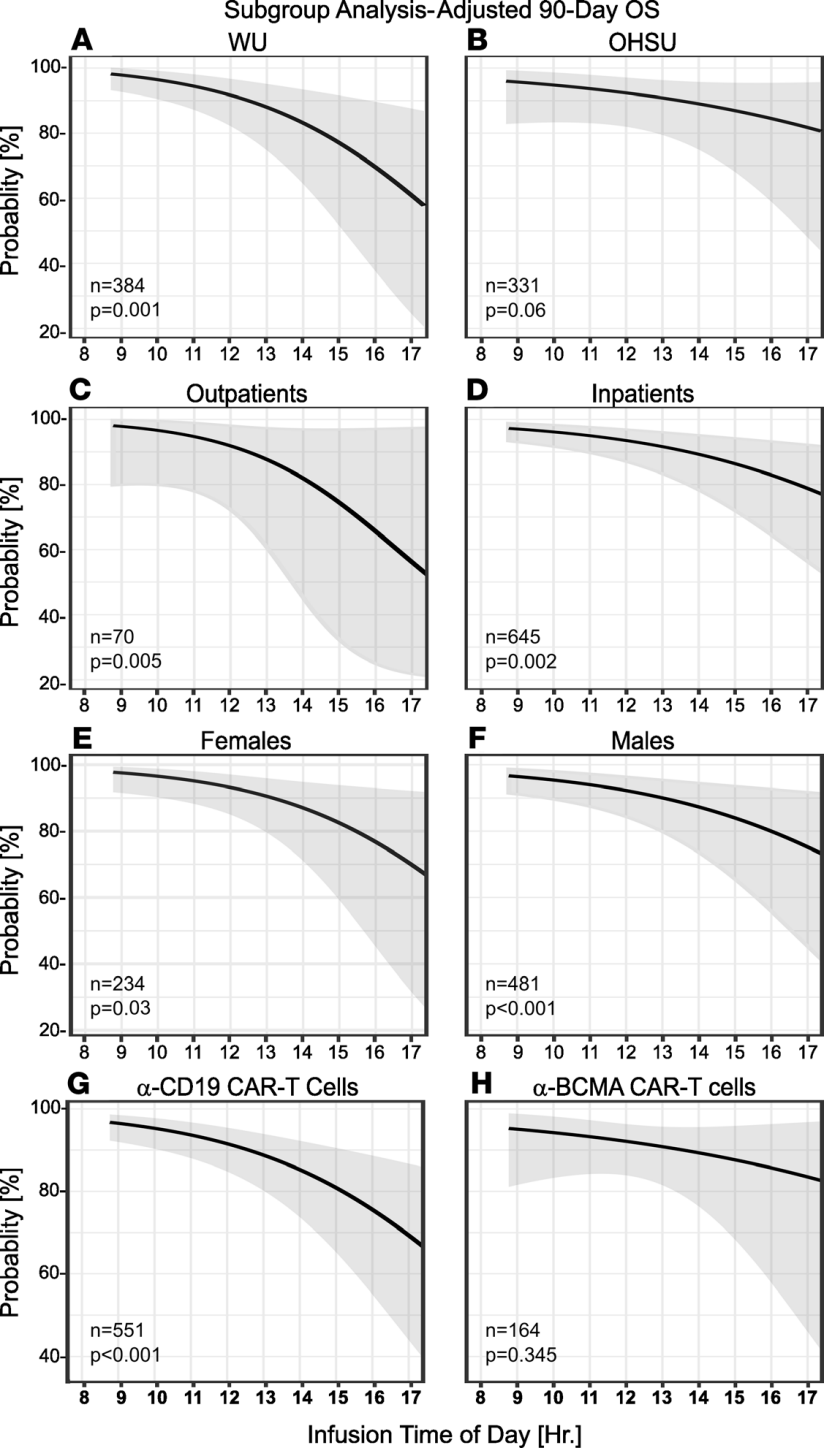
CAR-T cell outcomes are more time-sensitive in patients receiving anti-CD19 T cells. The graph depicts prespecified subgroup analyses using multivariable mixed-effects logistic regression, relating CAR-T infusion time to 90-day OS. (**A**–**H**) The subgroup analyses included location [WU (**A**) versus OHSU (**B**)], treatment setting [outpatient (**C**) versus inpatient (**D**)], sex [female (**E**) versus male (**F**)], and CAR-T cell target [CD19 (**G**) versus BCMA (**H**)]. Shaded areas represent 95% CIs. For each panel the Wald test *P* value for trend is depicted. The Supporting Data Values file contains this subgroup analysis expressed as an average hourly aOR similar to Figure 3.

Finally, we performed subgroup analyses of treatment location (WU versus OHSU), setting (inpatient versus outpatient), sex, and CAR T cell target (Figure 5). The inverse relationship between CAR T cell infusion time and survival was roughly similar across all subgroups (Figure 5, A–H). While OHSU subgroup achieved only a borderline *P* value (Figure 5B), this is likely because this location had a narrower range of infusion times than WU, with fewer early-morning and late-evening infusions that drive the effect (Supplemental Figure 1). The slope of the survival-infusion time relationship was qualitatively steeper for outpatients than for inpatients (Figure 5, C and D) and for females than for males (Figure 5, E and F). Patients receiving BCMA-targeted cells for multiple myeloma did not show a significant diurnal rhythm in 90-day OS (Figure 5H). Still, they showed a similar trend to CD19-targeted products, both in 90-day OS and in adjusted risk of severe ICANS (Supplemental Figure 7).

## Discussion

In this multicenter study using real-world patient data, we found a strong association between CAR T cell infusion timing and clinical outcomes, with earlier infusions associated with better survival and lower rates of severe neurotoxicity. The clinical effect of our findings may be substantial for patients and healthcare systems if treatment time proves to be causal. For example, each hour delay in CAR T cell infusion was associated with a 6%–8% average decrease in 365-day OS and EFS in our adjusted models (one additional adverse outcome per 13–17 patients). Thus, the hazards of giving CAR T cells later in the day are potentially on par with delaying antibiotics in septic shock or revascularization in myocardial infarction (11, 12). In those other scenarios, there was a shift to time-sensitive clinical practices (minimizing time to treatment). With these examples as precedent, our findings should prompt prospective trials evaluating time-of-day prioritization strategies to improve CAR T cell performance.

Our results also have implications for clinical trial design, particularly in evaluating new CAR T cell products and new indications, such as solid tumors, autoimmune disorders, and interstitial lung disease. Without controlling for time of treatment, diurnal rhythms in CAR T cell effectiveness could be a major confounder that leads to false-negative or false-positive conclusions. This mechanism has been postulated for the translational failure of some novel stroke treatments (13). Indeed, because trial protocols often require additional preinfusion procedures, coordination, or regulatory steps, investigational CAR T cell treatments may be more likely to occur later in the day than their standard-of-care counterparts, potentially obscuring the efficacy of novel therapies.

One potential challenge to implementing CAR-T cell chronotherapy involves operational inefficiencies caused by compressing treatments into a narrower interval of the day. However, these inefficiencies might be offset by improvements in patient outcomes and reductions in costly interventions such as rescue therapies like anakinra. Furthermore, subgroup analysis implied that prioritizing women or patients receiving anti-CD19 treatments for early-morning infusions could be especially beneficial. These subgroup patterns also raise important biological questions. The observation that CAR-T cell performance may be more time-sensitive in women was also seen in analogous studies of checkpoint inhibitor immunotherapies (14–16). This difference warrants further investigation, with particular attention to premenopausal versus postmenopausal women, given that circadian factors interact with sex hormones (17).

Why anti-CD19 CAR-T cell performance might be more time sensitive is unclear. Since these cells are given to treat lymphomas and leukemias, while anti-BCMA CAR-T cells are used for multiple myeloma, we speculate that tumor-specific factors are responsible. Specifics of CAR-T cell product design and baseline patient risk could also play a role.

We observed that the risk of severe ICANS (and corresponding anakinra use) increased with later administration times of CAR T cells. In contrast, neither severe CRS nor tocilizumab use varied significantly after adjustment for covariates. Several biological mechanisms specific to neurotoxicity could explain this finding. For example, blood-brain barrier permeability varies diurnally in preclinical models, which might affect the ability of CAR T cells to invade the CNS (18). Alternatively, circadian rhythms intrinsic to microglia or astrocytes could provoke differing levels of neuroinflammation upon encountering infiltrating CAR T cells (19). More research is needed to investigate these possibilities.

This study has several strengths, including 2 geographically distinct treatment centers, consideration of adverse effects and survival outcomes, complementary analytical approaches, and sensitivity analyses. There are also study limitations to consider. As with any retrospective study, the possibility of unmeasured confounding, including confounding by indication, can never be entirely excluded. Nonetheless, patients receiving late-day CAR T administrations had similar performance status, comorbidity burden, and illness severity compared with patients receiving earlier infusions, and we explicitly adjusted for observable preinfusion differences (e.g., time in hospital). E-values indicate that substantial unmeasured confounding would be required to nullify our findings (20). While our data estimate associations between time of day and CAR T cell performance at a population level, it cannot estimate individual patient risks. This is because biological rhythms in any individual can vary with lifestyle factors such as chronotype (e.g., habitual early versus late risers) and night shift work. A precision approach leveraging actigraphy or other patient-specific data to estimate optimal treatment times may help further mitigate risk. Although the sample size of 715 patients limited our ability to evaluate long-term, rare, or emerging adverse CAR T cell outcomes (e.g., CAR^+^ peripheral T cell lymphomas) (21), it is nonetheless sizable for this relatively new therapy. The limited observational window of our study could miss late-onset complications of CAR T cell therapy like protracted cytopenia (also called immune effector cell associated hematotoxicity [ICAHT]) (22). Further research will be needed to consider this and to examine whether the timing of lymphodepletion chemotherapy prior to CAR T cell treatment could contribute to the risk of ICAHT.

Finally, although we observe time-of-day variations in CAR-T clinical effectiveness, we cannot prove these are a direct effect of the circadian clock. However, preclinical evidence supports circadian clock involvement, and the observed morning advantage in CAR T cell outcomes aligns with predictions based on animal models (9). The fact that the time-outcome relationships we observe become stronger when time of day is expressed relative to sunrise suggests the presence of a circadian rhythm. More broadly, the convergence of multiple unrelated immunotherapies — spanning vaccines, immune checkpoint inhibitors, HSCT, and now CAR T cells — onto time-of-day patterns in which mornings are clinically optimal further suggests circadian biology may play a fundamental role. In conclusion, our analyses identify the time of day as a compelling and easily modifiable candidate factor for optimizing CAR T cell clinical performance, which should be evaluated in future prospective trials.

## Methods

### Sex as a biological variable.

Our study examined male and female patients.

### Study design and setting.

This is a retrospective cohort study of all adult patients receiving a single treatment of CAR T cells as a standard-of-care therapy for a hematologic malignancy at 2 NCI-designated Comprehensive Cancer Centers: Siteman Cancer Center at WU School of Medicine, and Knight Cancer Institute at OHSU between January 1, 2018, and March 31, 2025. Relevant to circadian rhythms, which are sensitive to light-dark cycles, the 2 centers are situated at different latitudes (38.6°N for WU, 45.5°N for OHSU).

Indications for CAR T cell treatment included diffuse large B cell lymphoma, mantle cell lymphoma, follicular lymphoma, acute lymphocytic B cell leukemia, and multiple myeloma. There were 6 FDA-approved CAR T cell products used during the study period: axicabtagene ciloleucel (axi-cel), lisocabtagene maraleucel (liso-cel), tisagenlecleucel (tisa-cel), brexucabtagene autoleucel (brexu-cel), ciltacabtagene autoleucel (cilta-cel), and idecabtagene vicleucel (ide-cel).

During the study period, WU exclusively administered CAR T cell therapy in the inpatient setting, while OHSU performed some infusions in the outpatient setting for selected patients (23). At both institutions, the timing of CAR T cell administration primarily depends on the pharmacy logistics and clinical staffing (e.g., attending physician availability).

### Study period.

The study period ranged from January 1, 2018, to March 31, 2025. All patients had at least 90 days of follow-up after treatment. We analyzed data between March and September of 2025.

### Study population, data sources, and organization.

We extracted electronic health records (EHR; Epic) of all adult patients receiving a single treatment of CAR T cells as a standard-of-care therapy for a hematologic malignancy (Figure 1). These included demographic and encounter data, vital signs, laboratory and microbiology tests, and medications. All CAR T infusions had documented timestamps. For variable definitions and missingness, see Supplemental Table 1. For the STROBE checklist, see Supplemental Table 9.

### Study outcomes.

The primary outcome was 90-day OS. Secondary outcomes were: 90-day EFS (defined based on relapse, progression, or death from any cause), 365-day OS and EFS (among patients with at least 1 year of observation time), the occurrence and timing of disease progression or relapse, development (and grade) of CRS or ICANS (based on American Society for Transplantation and Cellular Therapy consensus grading), new infection (defined as newly positive blood, respiratory, or urine culture, newly positive respiratory viral test, or newly positive test for *Clostridium difficile* infection within 14 days of CAR T cell administration), intensive care unit (ICU) admission, invasive mechanical ventilation (IMV), and shock (receipt of vasoactive medications) (24, 25). In calculating survival measures from the time of CAR T cell infusion, we censored patients who were alive and event-free at last follow-up or on April 1, 2025 (whichever came first).

### Statistics.

The primary exposure was time of day, modeled as a continuous variable. We analyzed infusion times based on hours after local sunrise (to adjust for latitude differences between the centers, seasonal changes to photoperiod, and daylight saving time) (26). This adjustment addresses biologic plausibility since circadian rhythms are strongly synchronized by sunlight. To facilitate categorical comparisons in time-to-event analyses, we dichotomized time at 15:00. We chose this threshold based on practical relevance, as 15:00 approximates the midpoint of afternoon and early evening clinical activities (12:00–18:00). This time is also consistent with studies of immune checkpoint inhibitor and HSCT effectiveness as a function of infusion times (8, 15, 27). We present data as *n* (%) and median (interquartile range [IQR]). We used χ^2^ and Wilcoxon rank-sum tests for unadjusted comparisons, as appropriate.

In the main analysis, we used multivariable mixed-effects logistic regression models (with hospital as a random effect) to determine the adjusted relationship between time of day and dichotomous outcomes; each model yielded an adjusted odds ratio corresponding to each additional hour later in CAR T cell administration. To enhance clinical interpretability of the landmarked survival models, we calculated the absolute risk reduction (ARR) at each hour (relative to local sunrise), summarizing the mean ARR over this time window and obtaining confidence intervals via nonparametric bootstrap.

All multivariable models were adjusted for covariates selected in advance based on a directed acyclic graph informed by the literature, biological plausibility, and our clinical experience (Supplemental Table 2) (28). These included age, sex, diagnosis indicating CAR T therapy, lymphodepletion regimen, Eastern Cooperative Oncology Group (ECOG) performance status, comorbidity burden (van Walraven–weighted Elixhauser index), severity of illness on day 0 (Simplified Acute Physiology Score II [SAPS-II]), CAR T cell dose, serum lactate dehydrogenase (LDH) as a surrogate for tumor burden, temporal trends (season and calendar year within study), and time in hospital before infusion (defaulting to 0 days for outpatient administrations) (29–31).

Concurrently, we used Kaplan-Meier methods to estimate survival distributions for early and late CAR T cell administrations and compared them using log-rank tests. To quantify absolute survival differences over a fixed time horizon, we compared each group’s 2-year RMST (32). We then fit multivariable survival models to account for possible confounding. We initially used multivariable Cox models with hospital-clustered robust standard errors; however, Schoenfeld residuals indicated proportional hazards assumption violations (*P* < 0.01). We therefore estimated covariate-adjusted 2-year RMST via the pseudo-observation approach; this approach yields an exposure regression coefficient that directly represents the adjusted difference in 2-year RMST per hour change in CAR T cell administration time (33). For these models, we obtained confidence intervals via parametric bootstrap to account for model uncertainty and the small number of hospital clusters.

For each model in the main analysis, we calculated E-values for the average adjusted ORs achieving statistical significance (Figure 3 and Supplemental Table 3) to quantify the robustness of effect estimates to hypothetical unmeasured confounders (20). The E-value is an odds ratio representing the minimum strength of association that an unmeasured confounder would need to have (with both the exposure and the outcome) to negate the observed association.

We performed 4 prespecified subgroup analyses. First, we compared the 2 treatment locations (WU and OHSU). Second, we compared inpatient versus outpatient CAR T cell administrations; the latter were prescheduled and predominantly for low-risk patients, making it less likely that acute patient factors would influence time-outcome relationships (23). Third, since circadian patterns are sex-dimorphic, we stratified our analyses according to this variable (17). Fourth, we compared results for patients receiving B cell maturation antigen–directed (BCMA-directed) versus CD19-directed CAR T cells, hypothesizing that tumor- or treatment-specific factors may modify temporal effects.

We analyzed patterns in commonly used biomarkers based on the timing of CAR T cell administration. We compared day 0, day 7, day 10, and day 14 ALCs, as well as peak values (within 14 days) of serum C-reactive protein (CRP), LDH, and ferritin measurements between early (before 15:00) and late administration times. At OHSU, where additional biomarkers were routinely available from clinical care, we made analogous comparisons of IFN-γ, TNF-α, IL-1, IL-2, IL-5, IL-6, IL-7, IL-10, IL-12, and IL-13.

We regarded *P* < 0.05 as statistically significant. All analyses used R 4.4.1 (R Project for Statistical Computing) and the following packages: tidytable, collapse, gtsummary, ggplot2, pseudo, lme4, and EValue.

### Study approval.

The IRBs at WU (no. 202410178) and OHSU (no. 00027601) approved this study. Since this was a retrospective study, patient consent was not required. See the online supplement for STROBE reporting documentation (Supplemental Table 9).

### Data availability.

R code for all analyses is available at https://github.com/p-lyons/017_car-t (commit ID a045ac4). In accordance with WU and OHSU data-sharing policies, deidentified data collected for the study might be made available upon approval by the study investigators, with relevant data-sharing agreements and ethics approvals. Requests should be directed to the corresponding author. See the Supporting Data Values and supplemental files for tabular presentation of data visualized in the figures.

## Author contributions

CAM, PGL, and JAH conceived the study. EG, MB, PK, BPE, ZAL, KML, and BHL extracted the data. PGL, JAH, CLH, NS, GH, JMH, CAM, and HM performed analyses and interpreted the data. PGL, CAM, HM, and JAH composed the manuscript. All authors vouch for the data and analysis. All authors participated in the editing and critical review of the manuscript and decided to submit it for publication.

## Funding support

This work is the result of NIH funding, in whole or in part, and is subject to the NIH Public Access Policy. Through acceptance of this federal funding, the NIH has been given a right to make the work publicly available in PubMed Central.

- NIH K08CA270383 (PGL)
- NIH R01HL172823 (JAH)
- NIH R01HL173976 (JAH)
- Department of Internal Medicine, WU School of Medicine (CAM)

## Supplementary Material

Supplemental data

ICMJE disclosure forms

Supporting data values

## Figures and Tables

**Table 1:**
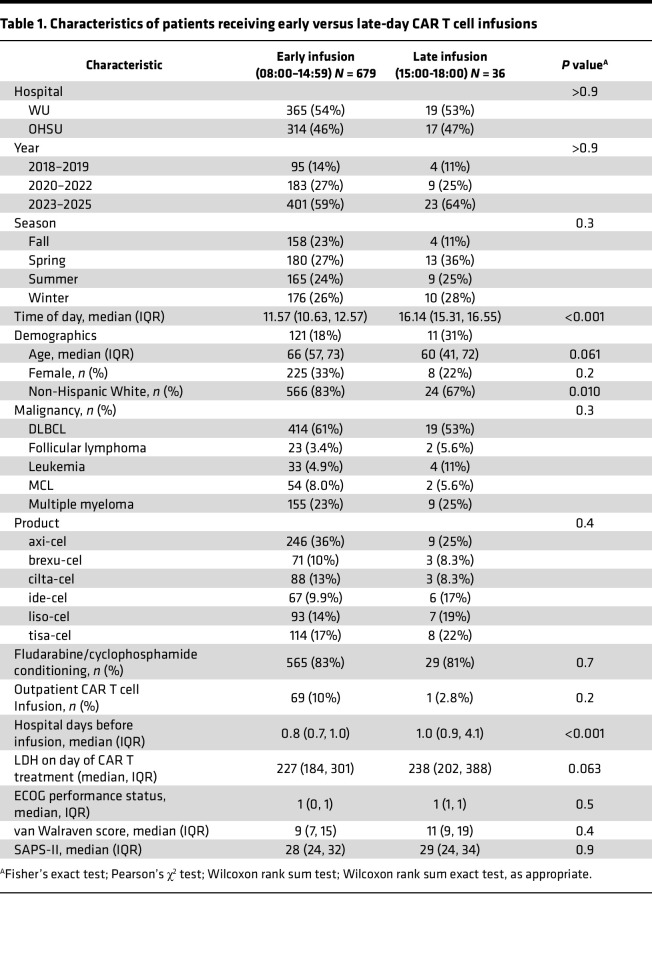
Characteristics of patients receiving early versus late-day CAR T cell infusions

**Table 2:**
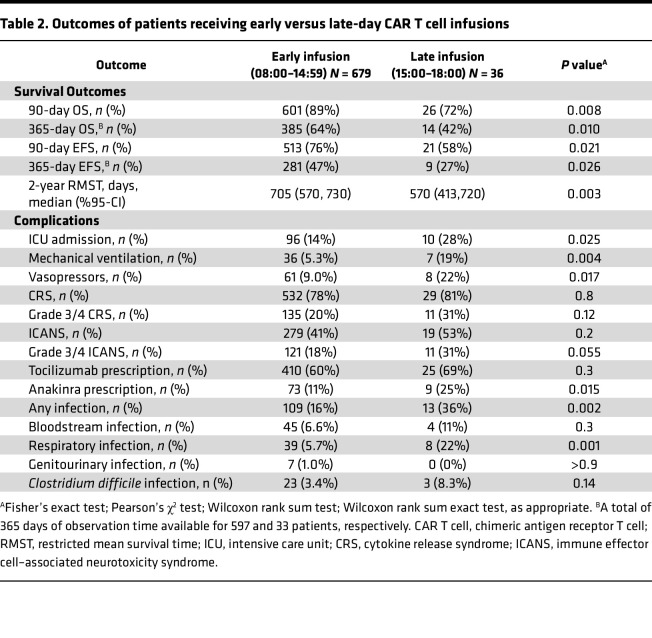
Outcomes of patients receiving early versus late-day CAR T cell infusions

## Version 1 12/18/2025

In-Press Preview

## Version 2 02/09/2026

Electronic publication
