# Supplementary material for: Diurnal rhythm in chimeric antigen receptor T cell effectiveness in an observational study of 715 patients
Source: JCI Insight. 2025 Dec 18;11(3):e201159. doi: 10.1172/jci.insight.201159 (PMC12892897; doi:10.1172/jci.insight.201159)
Supplement: Supplemental data [file jciinsight-11-201159-s104.pdf]

SUPPLEMENT

Diurnal Rhythm in Chimeric Antigen Receptor T-Cell Effectiveness in an Observational Study of 715 Patients

Patrick G Lyons, MD, MSc[1,2], Emily Gill[3], Prisha Kumar[3], Melissa Beasley[3], Brenna Park-Egan, MS[1], Zulfiqar A Lokhandwala[1], Katie M Lebold, MD, PhD[1], Brandon Hayes-Lattin, MD[2], Catherine L Hough, MD, MSc[1], Nathan Singh, MD, PhD[4,5], Guy Hazan, MD, PhD[6,7], Huram Mok, MD[3],Janice M Huss, PhD[3],Colleen A McEvoy, MD[3*], and Jeffrey Haspel, MD, PhD[3*]

[1]Division of Pulmonary, Allergy, and Critical Care Medicine, Oregon Health & Science University, Portland, OR, USA
[2]Knight Cancer Institute, Oregon Health & Science University, Portland, OR, USA
[3]Division of Pulmonary and Critical Care Medicine, Washington University School of Medicine, St. Louis, MO, USA
[4]Division of Oncology, Section of Cellular Therapy, Washington University School of Medicine, St. Louis, MO, USA
[5]Center for Gene and Cellular Immunotherapy, Washington University School of Medicine, St. Louis, MO, USA
[6]Faculty of Health Sciences, Ben Gurion University, Beer Sheva, Israel
[7]Pediatric Pulmonary Unit, Saban Children Hospital, Soroka University Medical Center, Beer Sheva, Israel

*Corresponding author contact information: Jeffrey Haspel, Division of Pulmonary and Critical Care Medicine, Department of Internal Medicine, Washington University School of Medicine, Campus Box 8052, 660 South Euclid Avenue, St. Louis, MO, 63110, USA. Email: jhaspel@wustl.edu

Conflict of interest statement: NS holds equity in Phoreus Bio, is a co-founder of Defiance Therapeutics, and has patents related to engineered T cell therapies, some of which have been licensed to Novartis and all of which are managed by the University of Pennsylvania or Washington University. All other authors declare no competing interests.

Supplemental Figure 1

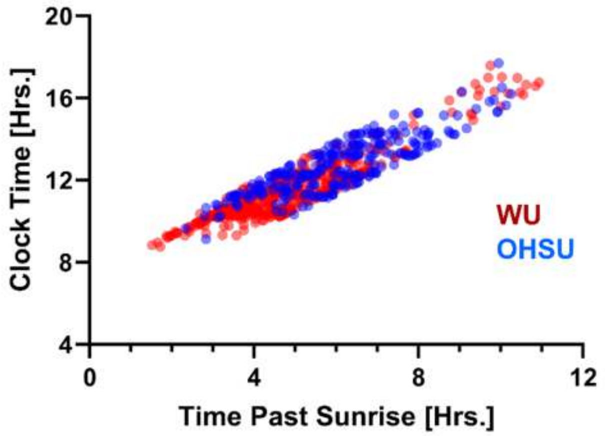

Supplemental Figure 1. Comparison of CAR T-Cell infusion times plotted as local “clock” time versus hours past sunrise (n=715). Red symbols, patients from WU; blue symbols, patients from OHSU. Note that any given clock time can vary by up to 2 hours relative to sunrise, depending on the season. Also note that the WU cohort encompasses a broader range of infusion times.

Supplemental Figure 2

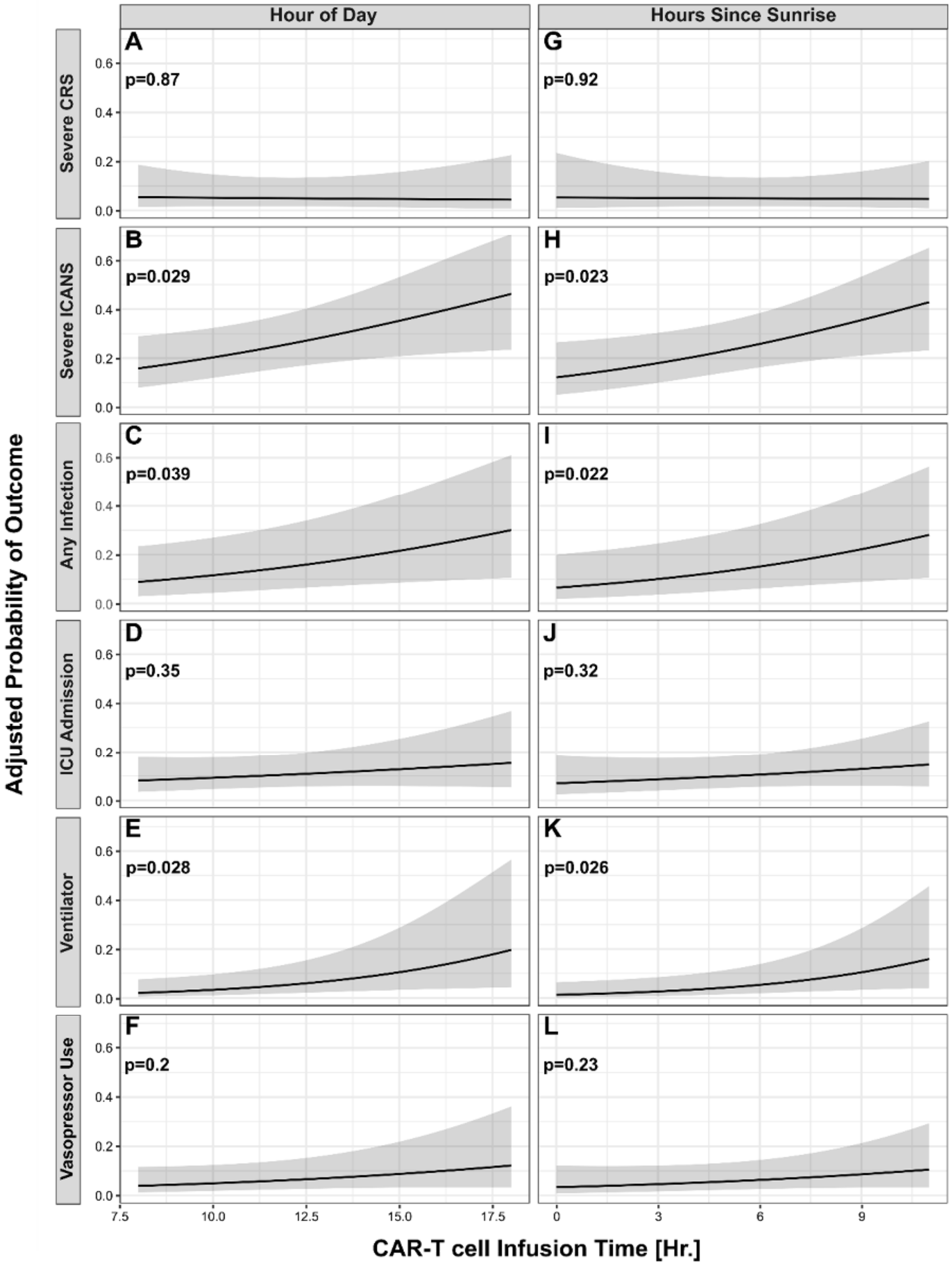

Supplemental Figure 2. Association between CAR-T cell infusion times and complications. The panels depict multivariate logistic regression analyses correlating the probability of selected complications with CAR-T infusion time, expressed as hours of the clock (**A-F,** left column) or hours since local sunrise (**G-L**, right column). Each row represents a different complication as labeled to the right. Shaded areas represent 95%-CIs. Wald test calculated p-values are depicted. p-values are derived from approximate Wald tests based on the estimated covariance of the spline coefficients.

Supplemental Figure 3

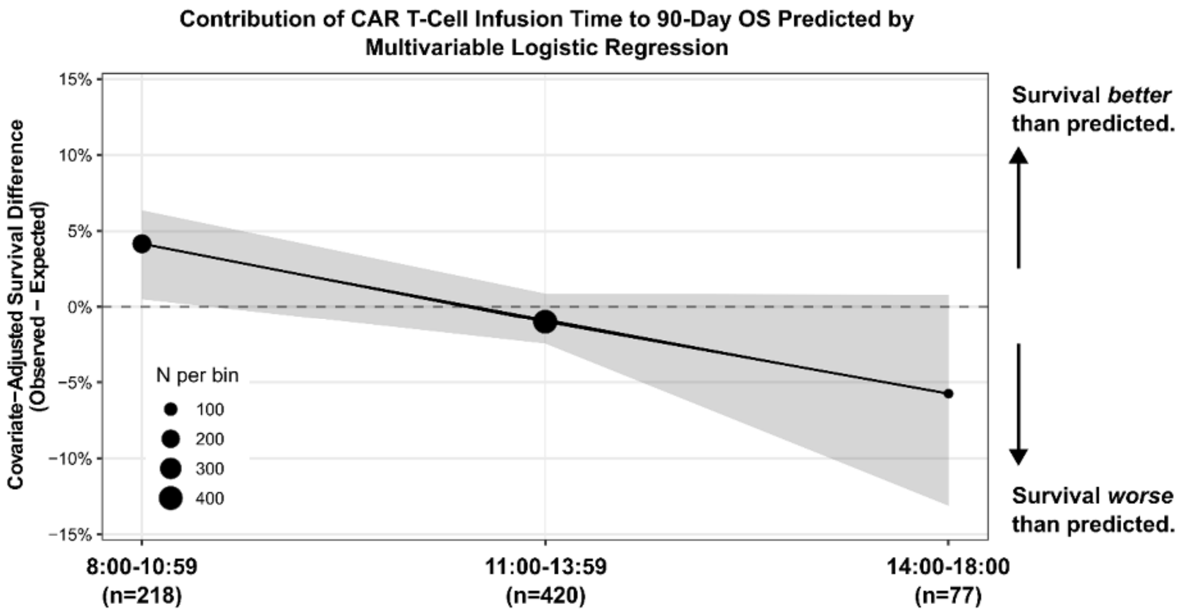

Supplemental Figure 3. Including time of day significantly improves logistic regression modeling of post-CAR T-cell survival. The plot depicts a covariate adjusted empirical risk as a function of CAR T-cell infusion time. It was generated by fitting our multivariable regression model as described in Methods but excluding infusion time. Patients were then grouped into 3 bins to generate via this model an "expected" 90-day OS. This was then subtracted from the observed 90-day OS to yield the covariate-adjusted survival difference depicted on the y-axis. Shaded areas represent 95%-CIs. Note that 90-day OS in the morning infusion bin "outperforms" the model by approximately 5% while late day infusions underperform by roughly 5%.

Supplemental Figure 4

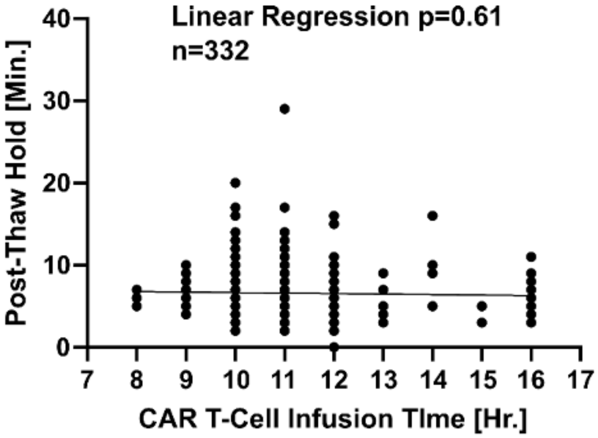

Supplemental Figure 4. No correlation between CAR-T cell Infusion time and the product post-thaw hold duration. Data were abstracted from WU EHR records where there was a timestamp indicating when the CAR-T cells began thawing (n=332, 86.5% of the total cohort). “Post-thaw hold” duration is defined as the interval between the start of thawing the CAR-T cells and the beginning of product administration to the patient. Significance was determined via linear regression, with the null hypothesis being that the slope of the regression line is zero (i.e. no relationship between infusion time and post-thaw hold duration).

Supplemental Figure 5

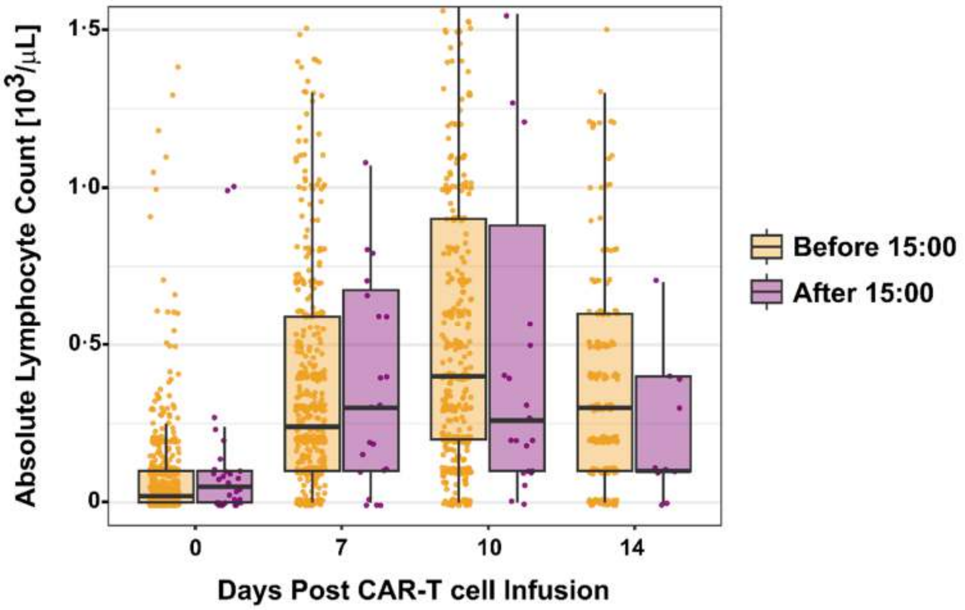

Supplemental Figure 5. Absolute lymphocyte count recovery did not substantially differ by CAR-T cell infusion time of day. The x-axis represents time, in days, since CAR T-cell infusion. The y-axis represents the absolute lymphocyte count. Orange indicates patients infused before 15:00 (n=384-673) and purple indicates those infused at or after 15:00 (n=17-30). Each value is one scattered point. Boxes represent the interquartile range (IQR) with the horizontal line indicating the median; whiskers extend to 1·5 × IQR.

Supplemental Figure 6

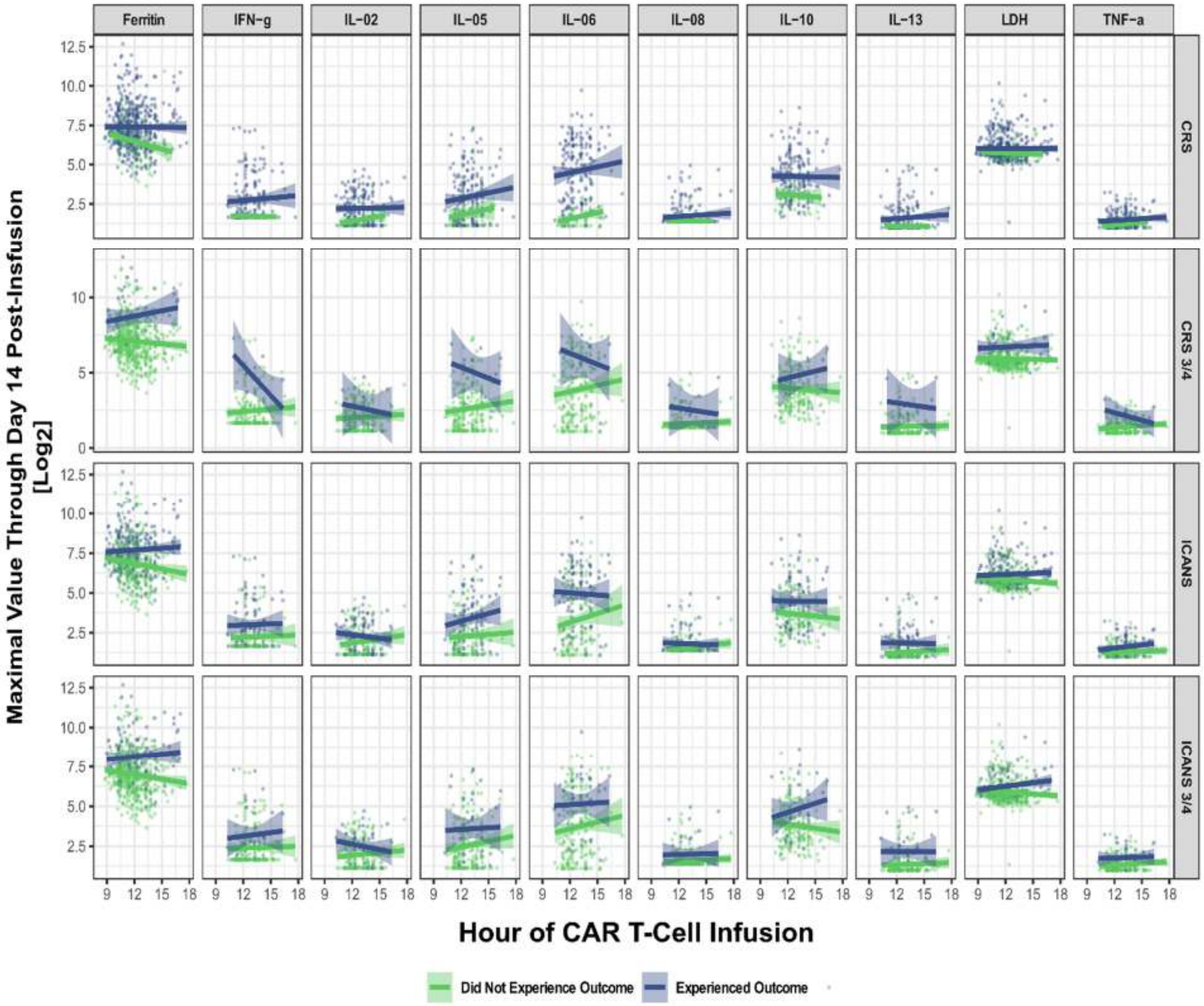

Supplemental Figure 6. Maximal laboratory values as a function of CAR-T Infusion time. Columns depict scatter plots of the maximal serologic levels of the indicated test out to day 14 post-CAR-T cell infusion (n=167-661). The data are expressed in log units (y axis) versus CAR T-cell infusion time (x-axis). Each row groups the data based on the occurrence of a specific complication indicated to the right of the panel. Trends lines ± 95%-CI are depicted within each panel. Blue lines represent the temporal trend amongst patients who experienced the specified outcome (e.g. CRS); green lines represent the trend for patients who did not experience the outcome.

Supplemental Figure 7

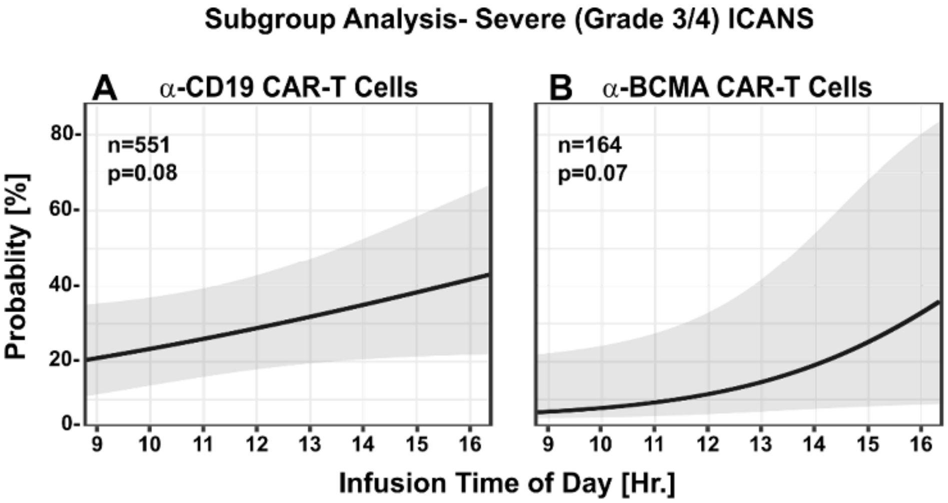

Supplemental Figure 7. Adjusted risk of severe ICANS binned by CAR target. Panels (**A**) and (**B**) depicted marginal effects plots from our multivariable logistic regression model for patients receiving CD19 (n=551) versus BCMA-directed products (n=164), respectively. Wald p-values for trend are depicted in each graph. Note that CD19-directed CAR T-cells are given for indications of lymphoma or leukemia, while BCMA-directed products are indicated for multiple myeloma.

Supplemental Table 1. Characteristics and outcomes of patients receiving CAR T-cell infusions at WU and OHSU.

Patient Characteristics	WU N = 384	OHSU N = 331	p Value
Age, years, median (IQR)	66 (58, 73)	65 (56, 72)	0.045
Female, n (%)	114 (29.7%)	119 (36.0%)	0.075
Non-Hispanic Caucasian, n (%)	334 (87.0%)	256 (77.3%)	<0.001
Malignancy, n (%)			<0.001
DLBCL	249 (64.8%)	184 (55.6%)	
Other Lymphomas	33 (8.6%)	48 (14.5%)	
Leukemia	16 (4.2%)	21 (6.3%)	
Multiple Myeloma	86 (22.4%)	78 (23.6%)	
Product			<0.001
axi-cel	188 (50.0%)	67 (20.2%)	
brexu-cel	33 (8.6%)	41 (12.4%)	
cilta-cel	50 (13.0%)	41 (12.4%)	
ide-cel	36 (9.4%)	37 (11.2%)	
liso-cel	42 (10.9%)	58 (17.5%)	
tisa-cel	35 (9.1%)	87 (26.2%)	
Hour of Day, median (IQR)	11:00 (10:24, 12:06)	12.24 (11:42, 13:42)	<0.001
Hours After Sunrise, median (IQR)	4.6 (3.6, 5.6)	5.9 (4.8, 6.9)	<0.001
Fludarabine/Cyclophosphamide Conditioning, n (%)	350 (91%)	244 (74%)	<0.001
Outpatient CAR T-cell Infusion, n (%)	< 5 (<1.5%)	66 (19.9%)	<0.001
Hospital Days Before Infusion, median (IQR)	0.8 (0.7, 3.1)	0.8 (0.6, 0.9)	<0.001
LDH on Day of Infusion, IU/L, median (IQR)	240 (203, 325)	206 (169, 277)	<0.001
ECOG Performance Status, median, IQR)	1 (0, 1)	1 (1, 1)	0.011
Van Walraven Comorbidity Score, median (IQR)	9 (9, 14)	12 (2, 19)	>0.9
SAPS-2, median (IQR)	29 (28, 32)	27 (24, 34)	0.010
Time Period, n (%)			0.002
2018-2019	64 (16.7%)	35 (10.6%)	
2020-2022	115 (29.9%)	77 (23.3%)	
2023-2025	205 (53.4%)	219 (66.2%)	
Season, n (%)			0.3
Fall	83 (21.6%)	79 (23.9%)	
Spring	113 (29.4%)	80 (24.2%)	
Summer	85 (21.8%)	89 (26.9%)	

Winter	103 (26.8%)	83 (25.1%)	
**Patient Outcomes**			
Overall survival, 90 days	335 (87.1%)	292 (88.2%)	0.7
Event-free survival, 90 days	280 (72.9%)	254 (76.7%)	0.2
Overall survival, 365 days*	230 (63.5%)	169 (63.1%)	>0.9
Event-free survival, 365 days*	164 (45.3%)	126 (47.1%)	0.7
ICU admission, n (%)	63 (16.4%)	43 (13.0%)	0.2
Mechanical ventilation, n (%)	29 (7.6%)	14 (4.2%)	0.062
Vasopressors, n (%)	46 (12.0%)	23 (6.9%)	0.023
Infections, n (%)	91 (23.7%)	31 (9.4%)	<0.001
CRS, n (%)			
Any CRS	310 (80.7%)	252 (76.1%)	0.014
Grade 3/4 CRS	30 (7.8%)	15 (4.5%)	0.072
ICANS, n (%)			
Any ICANS	172 (44.8%)	126 (38.1%)	0.069
Grade 3/4 ICANS	73 (19.0%)	59 (17.8%)	0.7
Tocilizumab, n (%)	242 (63.0%)	193 (58.3%)	0.2
Anakinra, n (%)	31 (8.1%)	51 (15.4%)	0.072

*WU N = 362, OHSU N = 268 patients with at least 365 days observation time
IQR, interquartile range; DLBCL, diffuse large B-cell lymphoma; CAR T-cell, chimeric antigen receptor; ECOG, Eastern Cooperative Oncology Group; ICU, intensive care unit; CRS, cytokine release syndrome; ICANS, immune cell-associated neurotoxicity syndrome

Supplemental Table 2: Data co-variates, preparation and missingness handling.

**Variable**	**Modeling Strategy**	Encounters with missing values, n (%)	Value Imputed
Age	Continuous	0	NA
Sex	Dichotomous: Male, Female	0	NA
Race/Ethnicity	Dichotomous: Non-Hispanic White, All Others	17 (0.02%)	Modal value by hospital
Cancer Diagnosis	Categorical: Multiple Myeloma, Acute Leukemia, Lymphoma	0	NA
Lymphodepletion regimen	Dichotomous: Fludarabine/Cyclophosphamide, Other	0	NA
Lactate dehydrogenase	Continuous	34 (4.8%)	Median value by hospital
Performance status	Continuous	9 (0.01%)	Median ECOG by hospital, malignancy, and CAR T-cell product
Van Walraven Score	Continuous	71 (10%)	Median value by hospital
SAPS-2	Continuous, with age component excluded in models	0	NA
CAR T-cell dose	Continuous standardized by malignancy, product, and body weight	186 (26%)	Median dose by hospital, malignancy, and CAR T-cell product
Season	Categorical: Spring, Summer, Fall, Winter	0	NA
Time Period	Categorical: 2018-2019, 2020-2021, 2022-2024	0	NA
Days in hospital prior to CAR T-cell infusion	Continuous, defaulting to 0 for outpatient administrations	0	NA

Supplemental Table 3: Adjusted odds ratios and E-values associated with one-hour shifts in CAR T-cell infusion times.

Time Unit	Outcome	Avg. Adj. OR per Hour	95% CI	p Value	E Value
**Clock Time (Hrs.)**	OS, 90 Day	0.76	0.64-0.89	<0.001	1.57
	OS, 365 Day	0.86	0.76-0.97	0.014	1.38
	EFS, 90 Day	0.89	0.79-1.01	0.068	
	EFS, 365 Day	0.87	0.74-1.03	0.101	
	Grade 3-4 CRS	0.98	0.75-1.27	0.869	
	Grade 3-4 ICANS	1.16	1.02-1.33	0.029	1.37
	Tocilizumab Use	1.02	0.91-1.14	0.706	
	Anakinra Use	1.26	1-1.57	0.048	1.49
	Vasopressor Use	1.13	0.93-1.36	0.199	
	Infection	1.16	1.01-1.34	0.039	1.37
	Mechanical Ventilation	1.28	1.03-1.58	0.028	1.51
	ICU admission	1.07	0.92-1.25	0.352	
**Hours Past Sunrise**	OS, 90 Day	0.76	0.65-0.89	<0.001	1.56
	OS, 365 Day	0.85	0.75-0.95	0.007	1.39
	EFS, 90 Day	0.87	0.77-0.98	0.024	1.35
	EFS, 365 Day	0.86	0.74-1.00	0.045	1.37
	Grade 3-4 CRS	0.99	0.77-1.27	0.924	
	Grade 3-4 ICANS	1.17	1.02-1.33	0.023	1.37
	Tocilizumab Use	1.01	0.91-1.13	0.858	
	Anakinra Use	1.24	1.03-1.48	0.021	1.46
	Vasopressor Use	1.12	0.93-1.34	0.227	
	Infection	1.17	1.02-1.34	0.022	1.38
	Mechanical Ventilation	1.27	1.03-1.57	0.026	1.51
	ICU admission	1.08	0.93-1.25	0.317	

Supplemental Table 4: Multivariable logistic regression “leave one out” sensitivity analysis, using 90-day OS as an endpoint.

Time Unit	Hour Left Out	n	Avg. Adj. OR per Hour	95%-CI	p Value
**Clock Time (Hrs.)**	8	3	0.76	0.64-0.89	0.001
	9	32	0.74	0.62-0.88	0.001
	10	183	0.75	0.61-0.91	0.005
	11	184	0.76	0.63-0.90	0.003
	12	141	0.75	0.63-0.88	0.001
	13	95	0.77	0.64-0.91	0.002
	14	41	0.75	0.62-0.88	0.001
	15	17	0.74	0.62-0.87	0.001
	16	15	0.83	0.68-1.01	0.063
	17	4	0.76	0.64-0.90	0.002
**Hours Past Sunrise**	1	8	0.77	0.65-0.90	0.002
	2	35	0.75	0.63-0.88	0.001
	3	128	0.75	0.62-0.89	0.001
	4	167	0.75	0.62-0.90	0.002
	5	160	0.73	0.61-0.87	0.000
	6	119	0.78	0.66-0.92	0.003
	7	47	0.77	0.65-0.91	0.002
	8	3	0.74	0.62-0.87	0.000
	9	32	0.76	0.63-0.91	0.003
	10	16	0.80	0.66-0.96	0.018

Supplemental Table 5: Causes of death within 90 days were generally related to malignancy or treatment complications.

Cause of Death	WU, n = 34	OHSU, n = 35
Infection	6	6
Toxicity	19	5
Malignancy	4	17
Other	5	7

Supplemental Table 6: Review of EMR-documented rationales for CAR T-cell administration after 15:00.

**Documented rationale for late infusion**	**N**
Infection workup	4
Hospital logistics (e.g., delays obtaining vascular access, limited clinician availability)	12
No clear reason documented	20

Supplemental Table 7: Time-to-event sensitivity analysis varying cutoff hour between early and late groups.

Early/Late<br><br>Cutoff Hour	N Early	N Late	Increase in 2-Yr. RMST with Early Infusions (Days ± 95% CI)	p Value
12:00	387	308	53.33<br><br>(8.97-96.33)	0.015
12:30	486	209	44.31<br><br>(1.2-90.13)	0.056
13:00	524	171	15.02<br><br>(33.15-60.33)	0.581
13:30	583	112	48.51<br><br>(5.9-102.83)	0.091
14:00	619	76	75.09<br><br>(13.13-134.65)	0.020
14:30	655	40	127.07<br><br>(46.35-207.3)	0.003
15:00	660	35	134.73<br><br>(49.22-221.75)	0.003
15:30	672	23	157.8<br><br>(53.55-269.51)	0.004

Supplemental Table 8: Maximal laboratory values through day 14 after CAR-T cell treatments.

Parentheses represent 95%-CIs. P values were calculated by the χ^2 Test.

Laboratory Measurement	Early CAR T-Cell Infusions (Before 15:00)	Late CAR T-Cell Infusions (After 15:00)	p-value
C-reactive Protein, median (IQR)	105 (52-169)	107 (59-190)	0.5
Lactate Dehydrogenase	325 (257-459)	418 (285-724)	0.06
Ferritin	1155 (567-2989)	2032 (519-5963)	0.13
IL-6	50 (7-299)	60 (4-500)	> 0.9
TNF-alpha	2.3 (1.7-4.3)	1.9 (1.7-7.2)	0.9
Interferon-gamma	5 (4-18)	5 (4-30)	> 0.9

Supplemental Table 9: STROBE checklist.

Item	#	Recommendation	Page #	Relevant manuscript text
Title	1A	Indicate the study's design with a commonly used term in the title or the abstract	1	Observational cohort study
Abstract	1B	Provide in the abstract an informative and balanced summary of what was done and what was found	2	Abstract page
Background	2	Explain the scientific background and rationale for the investigation being reported	3	Introduction section
Objectives	3	State specific objectives, including any prespecified hypotheses	3	…hypothesizing that earlier administration times would correlate with better clinical outcomes and fewer or less severe complications
Methods				
Study Design	4	Present key elements of study design early in the paper	8	Retrospective cohort study
Setting	5	Describe the setting, locations, and relevant dates, including periods of recruitment, exposure, follow-up, and data collection	8	Hospitals, dates, etc.
Participants	6	Give the eligibility criteria, and the sources and methods of selection of participants. Describe methods of follow-up	9	Inclusion/exclusion criteria
Variables	7	Clearly define all outcomes, exposures, predictors, potential confounders, and effect modifiers. Give diagnostic criteria, if applicable	9	All variables described
Data Sources	8	For each variable of interest, give sources of data and details of methods of assessment (measurement). Describe comparability of assessment methods if there is more than one group	9	Data sources, follow-up
Bias	9	Describe any efforts to address potential sources of bias	10	Model adjustment
Study size	10	Explain how the study size was arrived at	8, Supplemental 1	Convenience sample
Quantitative variables	11	Explain how quantitative variables were handled in the analyses. If applicable, describe which groupings were chosen and why	Supplemental Table 2	Full details in table

Statistical Methods	12A	Describe all statistical methods, including those used to control for confounding	10-11	Models, comparisons
	12B	Describe any methods used to examine subgroups and interactions	11-12	Subgroup analyses
	12C	Explain how missing data were addressed	8, Supplemental Figure 1	Imputation strategies
	12D	If applicable, explain how loss to follow-up was addressed	9	Study Period section
Results				
Participants	13	Report numbers of individuals at each stage of study—eg numbers potentially eligible, examined for eligibility, confirmed eligible, included in the study, completing follow-up, and analysed	3, Supplemental 1	All details reported
Descriptive Data	14	Give characteristics of study participants (eg demographic, clinical, social) and information on exposures and potential confounders	3, Supplemental Table 1	
Outcome Data	15	Report numbers of outcome events or summary measures over time	3-6	All rates reported
Main Results	16	Give unadjusted estimates and, if applicable, confounder-adjusted estimates and their precision (eg, 95% confidence interval). Make clear which confounders were adjusted for and why they were included	3-6	Model results give categories of confounders, rationale, and results with 95% CI
Other Analyses	17	Report other analyses done	6	Subgroup, lab analyses
**Discussion**				
Key results	18	Summarise key results with reference to study objectives	6	First paragraph
Limitations	19	Discuss limitations of the study, taking into account sources of potential bias or imprecision. Discuss both direction and magnitude of any potential bias	8-9	Limitations paragraph
Interpretation	20	Give a cautious overall interpretation of results considering objectives, limitations, multiplicity of analyses, results from similar studies, and other relevant evidence	6-8	Full Discussion
Generalizability	21	Discuss the generalisability (external validity) of the study results	6-9	Discussion of other circadian patterns in immunotherapy, discussion of population-level patterns

				vs individual-level predictions
Funding	22		13	Disclosed in the Acknowledgements section.
